# Identification of Mammaliicoccus fleurettii as the source of a methicillin-resistant gene in a First Nation reserve lake in Manitoba, Canada

**DOI:** 10.1099/acmi.0.000861.v3

**Published:** 2024-11-22

**Authors:** Sabrin Bashar, Rakesh Patidar, Alvan Wai, Dawn White, George R. Golding, Annemieke Farenhorst, Ayush Kumar

**Affiliations:** 1 Department of Microbiology, University of Manitoba, Winnipeg, Manitoba, Canada; 2 Department of Medical Microbiology and Infectious Diseases, Max Rady College of Medicine, University of Manitoba, Winnipeg, Manitoba, Canada; 3 Bacterial Pathogens, AMR, and Wastewater Division, National Microbiology Laboratory Branch, Public Health Agency of Canada, Winnipeg, Manitoba, Canada; 4 Department of Soil Science, University of Manitoba, Winnipeg, Manitoba, Canada

**Keywords:** *Mammaliicoccus fleurettii*, methicillin resistance, mobile genetic elements, water quality

## Abstract

Our study aimed to identify the bacterial source of a previously detected mobile antibiotic-resistant gene, *mecA*, found in a lake that serves as a source to a water treatment plant operated by a First Nation reserve. Three methicillin-resistant presumptive *Staphylococcus* spp. isolated from the sample using selective media were verified as *mecA* positive by PCR. MALDI-TOF and whole-genome sequencing of each isolate confirmed that all three were *Mammaliicoccus fleurettii*. Antibiotic-resistant gene analysis of the assembled genomes predicted *mecA* with 99.7% sequence identity, and phylogenetic analysis grouped our three *mecA* genes with the *mecA* allele from a methicillin-resistant strain of *Staphylococcus aureus*. Identifying microbial species known to harbour mobile antibiotic-resistant elements can provide greater depth of information about drinking water, an especially essential need in First Nation reserves where water quality too frequently is poor.

**Impact Statement**

The global presence of antibiotic-resistant bacteria is a major public health concern. The transmission of antibiotic-resistant genes via mobile genetic elements is primarily talked about within pathogenic bacteria; however, non-pathogenic bacteria can also act as carriers and promote the movement of these resistant genes. The *mecA* gene confers methicillin resistance and is one of the most significant mobile resistance elements to *β*-lactam antibiotics (e.g., penicillin). Surface waters – including lakes, rivers and streams –are ecosystems that can act as a vehicle for bacterial movement. We discovered the presence of *mecA*-harbouring *Mammaliicoccus fleurettii*, a non-pathogenic bacterium, in a lake that provides drinking water to a remote First Nation reserve in Manitoba, Canada. This finding raises concern as the *mecA* mobile genetic element could potentially be transferred to staphylococcal pathogens within the water supply or distribution system. Our study highlights the need to proactively monitor potable water sources for non-pathogenic bacteria harbouring harmful mobile antibiotic-resistant genes.

## Data Summary

All data and protocols for the three presumptive *Staphylococcus* spp. confirmed as *Mammaliicoccus fleurettii* have been provided within the article or are available upon request. NCBI assembly accession numbers: SF 001: GCA_016808275.1; SF 002: GCA_018310175.1; SF 003: GCA_018310195.1.

## Introduction

Access to safe drinking water has been deemed a basic human right by the United Nations, essential for people’s well-being and dignity [1]. Poor water conditions in developing countries are a long-fought problem, but there is little public awareness of the poor water quality that affects people in developed countries. Canadian drinking water supplies are generally considered to be excellent [2]; however, water quality across many First Nation reserves is extremely poor [3], often prompting long-term drinking water advisories [4].

One of the major reasons for water advisories is the poor microbiological quality of the water [5]. We already documented this problem in First Nation reserves in Manitoba, Canada, where drinking water was found to have high levels of *Escherichia coli* despite the communities having access to water treatment plants; we also revealed the presence of antibiotic-resistant genes – considered an emerging water contaminant [6] – within the water supply of these communities [7]. One of the antibiotic-resistant genes identified was *mecA*, a clinically significant methicillin-resistant gene exemplified by methicillin-resistant *Staphylococcus aureus* (MRSA). The *mecA* gene encodes for a penicillin-binding protein and makes bacteria harbouring the gene less vulnerable to eradication upon exposure to most *β*-lactam antibiotics [8]. The *mecA* gene is commonly located on the staphylococcal cassette chromosome *mec* (SCCmec), a mobile genetic element that can also carry other antimicrobial-resistant genes, virulence determinants and genes that confer survival under stress [9].

In this study, we aimed to identify the bacterial source of the *mecA* gene that we previously discovered in a First Nation reserve’s lake water [7] from an area used as a source of water and for recreational activities. Such information can help guide and improve water monitoring protocols and assess potential health risks to the members of the community.

## Methods

### Community profile and water sampling

The water sample was collected in June 2019 (congruent with the sampling location and time of year in the initial study [7]) from a natural lake within a remote First Nation reserve in Manitoba, Canada, at an area frequented by the community. This area of the lake is also used as a source of water for the water treatment plant in the First Nation reserve. The water sample was collected using a standard method [10]. Sterile sampling bottles were opened just prior to filling; the sample was preserved with 0.003% sodium thiosulfate and stored at 4 °C or on ice packs. The water sample was processed immediately upon arrival at the laboratory, within 24 h of collection.

### Isolation of methicillin-resistant presumptive *Staphylococcus* spp.

Oxacillin screening for methicillin-resistant presumptive *Staphylococcus* spp. followed published guidelines [11] and was performed with the lake water sample, autoclaved tap water (negative control) and an *S. aureus* clinical isolate HA-MRSA 100697 (positive control). Briefly, 100 ml of each water sample was filtered through a 0.45 µm sterile polyethersulfone membrane (Pall Corporation). Each membrane was placed on a lysogeny broth (LB) agar plate [BD Difco LB Agar (Lennox); Fisher Scientific] supplemented with 6 µg ml^−1^ oxacillin (Millipore Sigma) [11] and incubated at 37 °C for 48 h. Colonies from the LB+oxacillin plates containing the lake water sample were presumptively identified as methicillin-resistant staphylococci and were sub-cultured onto mannitol salt agar (MSA; Oxoid) supplemented with 6 µg ml^−1^ oxacillin for 48 h at 37 °C. Three yellow-pigmented colonies from the MSA+oxacillin plate – designated SF 001, SF 002 and SF 003 – were selected for further propagation and analysis, including MIC testing using the Gram-positive GPALL1F AST plate on the Sensititre system (Thermo Fisher Scientific).

### Genotypic analysis of the bacterial isolates

Colony PCR [12] was used to screen the isolates for three genes: *Staphylococcus* species-specific *rpoB* [13], methicillin-resistant *mecA* [14] and *S. aureus*-specific *nuc* [15] (Table S1, available in the online version of this article). The PCR products were resolved on 1.3% agarose gels and visualized using an Axygen BL Gel Documentation System (Corning).

### MALDI-TOF analysis of the bacterial isolates

The peptide profile of each of the isolates was determined using the Bruker Daltonics Microflex MALDI-TOF mass spectrometer. Chemical extraction of each bacterial isolate was performed following the manufacturer’s protocol; *S. aureus* was included as an extraction control. The Bruker Bacterial Test Standard (#8255343) was used to calibrate the system. One microlitre of each extract was spotted onto an MSP 96-well polished steel plate in triplicate, dried at room temperature and then overlaid with 1 μl of a saturated solution of α-cyano-4-hydroxycinnamic acid (Millipore Sigma). Using flexControl software (v.3.4), 240 laser shots (60 Hz N_2_-Cartridge-Laser) were accumulated for each spectrum. Spectra were analysed using MALDI BioTyper Compass Explorer software (v.4.1) and compared against the latest spectral database (BDAL DB, 8468 MSPs). Scoring was based on Bruker Daltonic’s BioTyper algorithm.

### Whole-genome sequencing of the bacterial isolates and genomic analysis

Whole-genome sequencing was performed using genomic DNA purified from each of the three isolates using the Epicentre MasterPure Complete DNA and RNA Purification kit (Illumina) and prepared using the Nextera XT DNA Library Preparation kit (Illumina). Sequencing was performed using the Illumina MiSeq (150 bp paired-end reads). Sequence quality was assessed via FastQC [16], and *de novo* assembly was conducted using SPAdes (v.3.14.0) [17]. The quality of the assembled genomes was assessed using QUAST v.5.0.2 [18], and annotation was carried out using the NCBI Prokaryotic Genome Annotation Pipeline [19]. Each assembled genome sequence was submitted to the NCBI GenBank database. Antibiotic-resistant genes were predicted from the assembled genomes using the Comprehensive Antibiotic Resistance Database using RGI *main* under strict (>95% identity) and perfect (100% identity) parameters [20]. Phylogenetic relationships of the *mecA* genes were analysed in mega X [21] using the neighbour-joining method [22]; pairwise deletion removed ambiguous positions [23]. Genomic alignments were performed using Geneious Prime [24], BLASTn [25] and Easyfig [26]. All analyses were performed using default settings to ensure consistency across each analysis.

## Results and discussion

Contaminated drinking water is a commonly recognized source of gastrointestinal infections [27]. Previous studies by us have highlighted the presence of antibiotic-resistant genes in lakes as well as post-treated tap and cistern water samples from First Nation reserves in Manitoba, Canada [7,28]. Families in these communities rely on lake water for their livelihood as well as spiritual practices. The presence of antibiotic-resistant microbes and their genes in these waters warrants further collaboration with the community to assess risk. The goal here was to determine the bacterial source of the *mecA* gene that we previously found in a water sample from a First Nation reserve [7].

We isolated three colonies from MSA plates supplemented with oxacillin, a standard screening media for pathogenic *Staphylococcus* spp. [11]. The smooth, elevated colonies were yellow, indicating the fermentation of mannitol [29]. The three isolates showed resistance towards oxacillin (resistance breakpoint ≥4 µg ml^−1^) and penicillin (resistance breakpoint 0.254 µg ml^−1^) (Table 1) according to Clinical and Laboratory Standards Institute guidelines [30]. A positive PCR reaction for the *Staphylococcus* species-specific *rpoB* gene and the *mecA* gene highly suggested that the isolates were staphylococci; however, the *nuc* gene was absent, meaning that the isolates were not specifically *S. aureus* (Fig. S1).

**Table 1.** MICs of antimicrobials in the GPALL1F AST plate for the three *M. fleurettii* isolates from this study

Antimicrobials	SF 001	SF 002	SF 003
Ampicillin	0.5	0.5	0.5
Chloramphenicol	8.0	8.0	8.0
Ciprofloxacin	≤1.0	≤1.0	≤1.0
Clindamycin	≤0.5	≤0.5	≤0.5
Daptomycin	≤0.5	≤0.5	≤0.5
Dtest1	≤4.0	≤4.0	≤4.0
Dtest2	≤8.0	≤8.0	≤8.0
Erythromycin	≤0.25	≤0.25	≤0.25
Gentamicin	≤2.0	≤2.0	≤2.0
Levofloxacin	0.5	0.5	0.5
Linezolid	2.0	2.0	2.0
Moxifloxacin	0.5	0.5	0.5
Nitrofurantoin	≤32.0	≤32.0	≤32.0
Oxacillin+2% NaCl	>4.0	>4.0	>4.0
Penicillin	0.5	1.0	0.5
Quinupristin/dalfopristin	≤0.5	≤0.5	≤0.5
High-level streptomycin (1000 µg ml^−1^)	≤1000	≤1000	≤1000
Tetracycline	≤2.0	≤2.0	≤2.0
Tigecycline	≤0.03	0.06	≤0.03
Trimethoprim/sulfamethoxazole	≤0.5	≤0.5	≤0.5
Vancomycin	1.0	1.0	1.0

We then performed MALDI-TOF mass spectrometric analysis on protein extracts from each isolate and confirmed that the bacterium was not *S. aureus*. Whole-genome sequencing and 16S rRNA annotation of each assembled genome allowed us to identify our isolates as *M. fleurettii*. *M. fleurettii* is a recently reclassified species, once called *Staphylococcus fleurettii* [31], and believed to be the first point of emergence of the *mecA* gene [32,33]. It is most commonly associated with cattle, sheep, goat and camelids [34]; has been isolated from wild animals (rabbits [35], shrews, voles and mice [36]) and companion animals (dogs and cats [37]) and has also been linked with bovine mastitis [38]. Most animal-associated * M. fleurettii* isolates showed the presence of *mecA* [34,35, 37]. To our knowledge, our samples are the first Canadian lake-water-derived, *mecA*-carrying *M. fleurettii* isolates; superficial waters and wastewater have also been documented as sources of *mecA*-harbouring *M. fleurettii* [39].

**Fig. 1.**
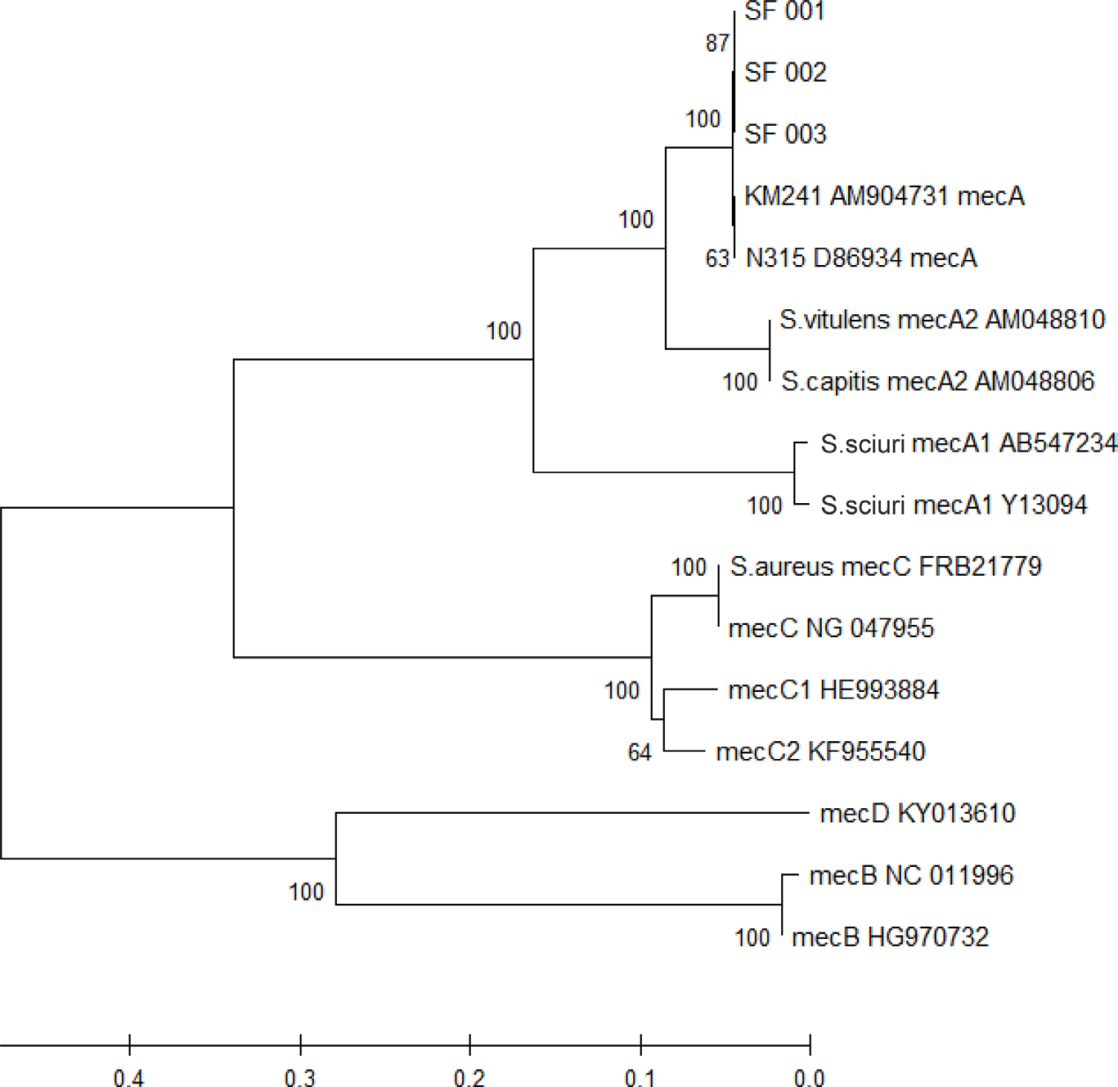
Phylogenetic analysis of the *mecA* gene from *M. fleurettii* (SF 001, SF 002 and SF 003) was inferred using the neighbour-joining method [22]. The optimal tree with the sum of branch length=1.373 is shown. Evolutionary distances were computed using the maximum composite likelihood method; branch lengths represent the number of base substitutions per site (scale shown), and units on the branches indicate branch support values; pairwise deletion removed ambiguous positions [23]. A total of 2042 positions comprised the final dataset (mega X [21]).

**Fig. 2.**
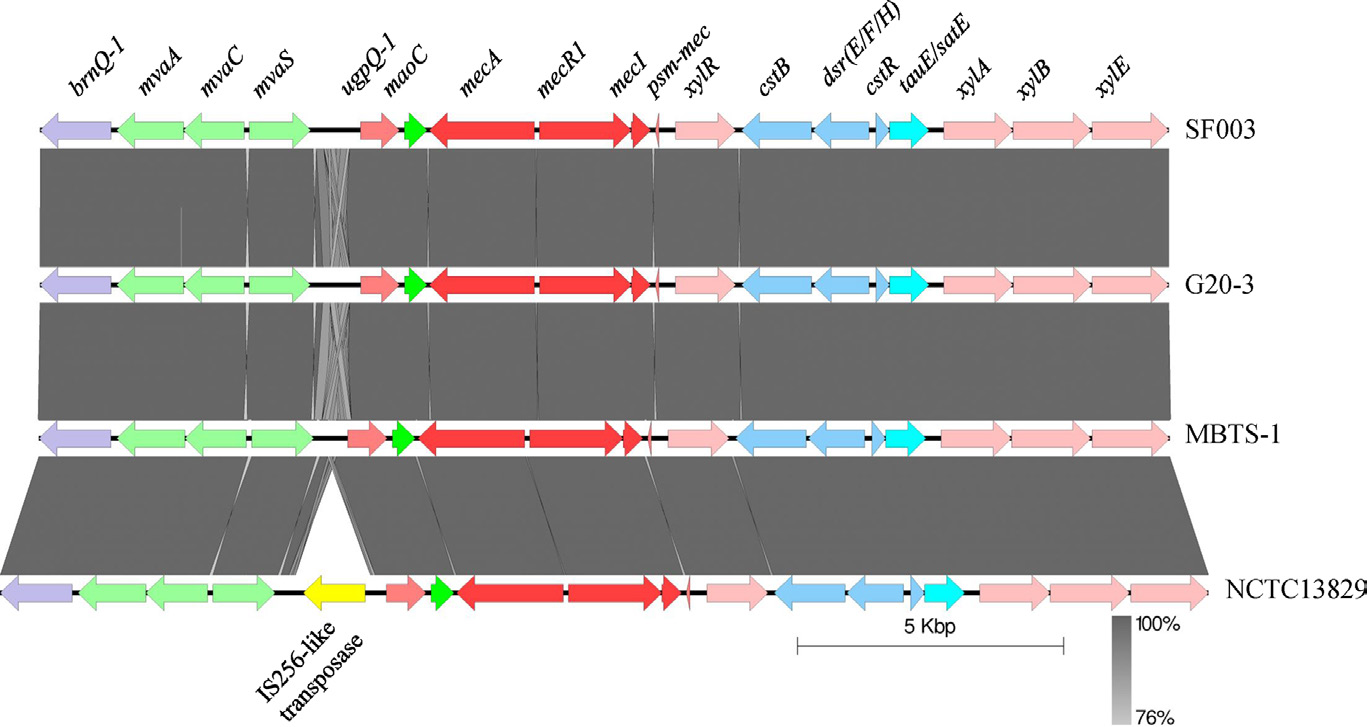
Genomic alignment of the *mec* gene complex and its flanking region using a representative *M. fleurettii* isolate (SF 003) along with a *M. fleurettii* reference genome (MBTS-1MBTS-1) and two other NCBI *M. fleurettii* genomes (G20-3 and NCTC18329G20-3NCTC18329) (Easyfig [26]).

Sequencing data were used to generate genome assemblies (total length: >2.47 Mbp; length N_50_: >113,000; %GC: 31%) (Fig. S2), identifying >2400 protein-encoding genes in each of the samples. Antibiotic-resistant genes were predicted from the assemblies using the Comprehensive Antibiotic Resistance Database; under ‘strict’ parameters, a hit yielding 99.7% sequence identity with *mecA* was found. No further antibiotic-resistant genes were identified under ‘loose’ parameters (<95% identity). The per cent nucleotide identity of the *me*c gene complex of our three isolates against reference genomes (MBTS-1, D86934.2MBTS-1D86934.2) showed at least 98.8% identity (Table 2). Phylogenetic tree analysis linked our three *mecA* genes to the *mecA* allele type from the SCCmec cassette of *Staphylococcus pseudintermedius* KM241 (AM904731.1AM904731.1) and methicillin-resistant *S. aureus* N315 (D86934.2D86934.2) (Fig. 1). Furthermore, the *mec* gene complex flanking region – a 21 881 bp sequence comprising *brnQ-1-mvaA-mvaC-mvaS-ugpQ-1-maoC-mecA-mecR1-mecI-psm-xylR-cstB-DsrE/DsrF/DsrH-cstR-TauE/SafE-xylA-xylB-xylE* – showed 100% nucleotide coverage and identity with a representative *M. fleurettii* isolate and three other *M. fleurettii* genomes (MBTS-1, G20-3MBTS-1G20-3 and NCTC18329NCTC18329) (Fig. 2).

**Table 2.** Per cent identity matrix of *mecA*, *mecR1* and *mecI* nucleotide sequences from an *M. fleurettii* reference genome (MBTS-1MBTS-1), a methicillin-resistant *S. aureus* N315 (D86934.2D86934.2) and the three *M. fleurettii* isolates from this study (SF 001, SF 002 and SF 003) (Geneious Prime [24])

Strains	MBTS-1 (%)	N315 (%)	SF 001 (%)	SF 002 (%)	SF 003 (%)
**MBTS-1**		98.82	98.96	98.96	98.96
**N315**	98.82		99.86	99.86	99.86
**SF 001**	98.96	99.86		100%	100%
**SF 002**	98.96	99.86	100%		100%
**SF 003**	98.96	99.86	100%	100%	

Our study had a defined purpose and so did not investigate peripheral questions. The source of *M. fleurettii* in this First Nation reserve’s lake water is unknown, but given the vastness and remoteness of the lake, it would be difficult for this to be precisely determined. One could speculate that *M. fleurettii* was of animal origin, wild or companion [35,37]. Given that we isolated *M. fleurettii* years after discovering the *mecA* gene does suggest, though, that there is a consistent source of contamination. Broader water sampling around the lake may be a beneficial future collaboration with the First Nation community.

## Conclusion

In this study, we showed that *M. fleurettii* was present in a lake water sample taken from a First Nation reserve in Manitoba, Canada, and established that this bacterium was a source of the *mecA* gene previously identified in the same water. The mobility of the *mecA* gene means that *M. fleurettii* can potentially transfer the gene to other known staphylococcal pathogens [40]. Our study underscores the importance of understanding the full bacterial composition of the lake and drinking waters to identify seemingly harmless bacteria harbouring transferable antibiotic-resistant genes. Such precautions may reduce potentially serious human health issues.

## supplementary material

10.1099/acmi.0.000861.v3 Uncited Supplementary Material 1.

## Abbreviations

LB: lysogeny broth
MRSA: methicillin-resistant *Staphylococcus aureus*
MSA: mannitol salt agar
SCCmec: staphylococcal cassette chromosome *mec*
