# Supplementary material for: Identification of Mammaliicoccus fleurettii as the source of a methicillin-resistant gene in a First Nation reserve lake in Manitoba, Canada
Source: Access Microbiol. 2024 Nov 22;6(11):000861.v3. doi: 10.1099/acmi.0.000861.v3 (PMC11652733; doi:10.1099/acmi.0.000861.v3)
Supplement: Uncited Supplementary Material 1. [file acmi-6-00861-s001.pdf]

Supplementary Information

Identification of *Mammaliicoccus fleurettii* as the source of a methicillin-resistance gene in a First Nation reserve lake in Manitoba, Canada

Sabrin Bashar[a], Rakesh Patidar[a], Alvan Wai[a], Dawn White[a], George R. Golding[b,c], Annemieke Farenhorst[d], Ayush Kumar[a#]

[a]Department of Microbiology, University of Manitoba, Winnipeg, Manitoba, Canada

[b]Department of Medical Microbiology and Infectious Diseases, Max Rady College of Medicine, University of Manitoba, Winnipeg, Manitoba, Canada

[c]Bacterial Pathogens, AMR, and Wastewater Division, National Microbiology Laboratory Branch, Public Health Agency of Canada, Winnipeg, Manitoba, Canada

[d]Department of Soil Science, University of Manitoba, Winnipeg, Manitoba, Canada

Table S1: PCR primers used in this study.

**Primer**	**Sequence (5′-3′)**	**Reference**
**rpoB forward**	CAATTCATGGACCAAGC	1
**rpoB reverse**	CCGTCCCAAGTCATGAAAC	
**mecA forward**	AACAGGTGAATTATTAGCACTTGTAAG	2
**mecA reverse**	ATTGCTGTTAATATTTTTTGAGTTGAA	
**nuc foward**	GCGATTGATGGTGATACGGTT	3
**nuc reverse**	AGCCAAGCCTTGACGAACTAAAGC	

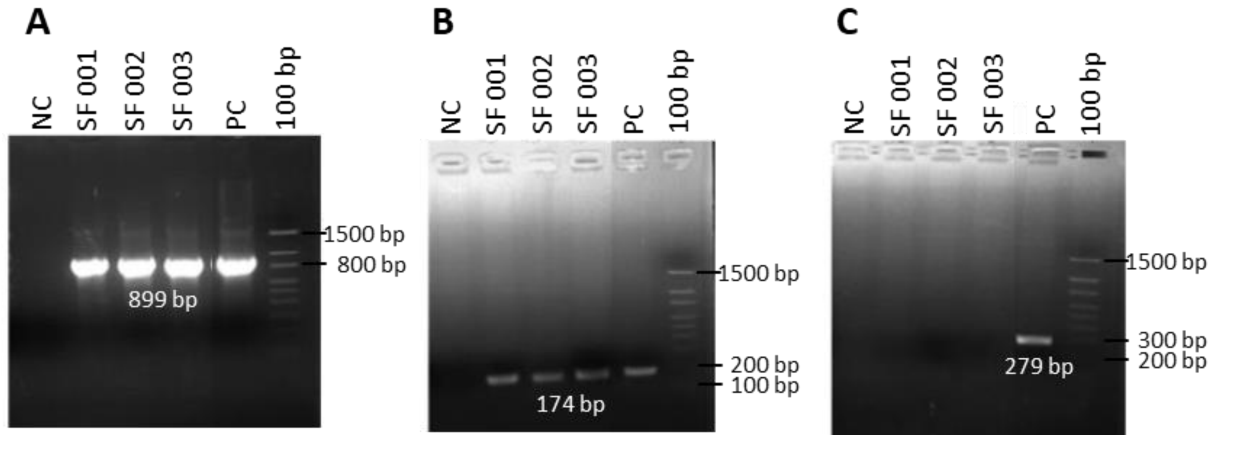

Figure S1: Agarose gel electrophoresis of PCR amplicons for genotypic analysis of bacterial isolates SF 001, SF 002, and SF 003. **(A)** *Staphylococcus* species-specific *rpoB* (RNA polymerase β subunit); **(B)** methicillin-resistance *mecA* (penicillin binding protein 2a); **(C)** *S. aureus*-specific *nuc* (thermonuclease). All gels from left to right: NC - negative control (no DNA template); SF 001; SF 002; SF 003; PC - positive control (*S. aureus* HA-MRSA 100697 genomic DNA); 100-base pair (bp) molecular marker (Biobasic).

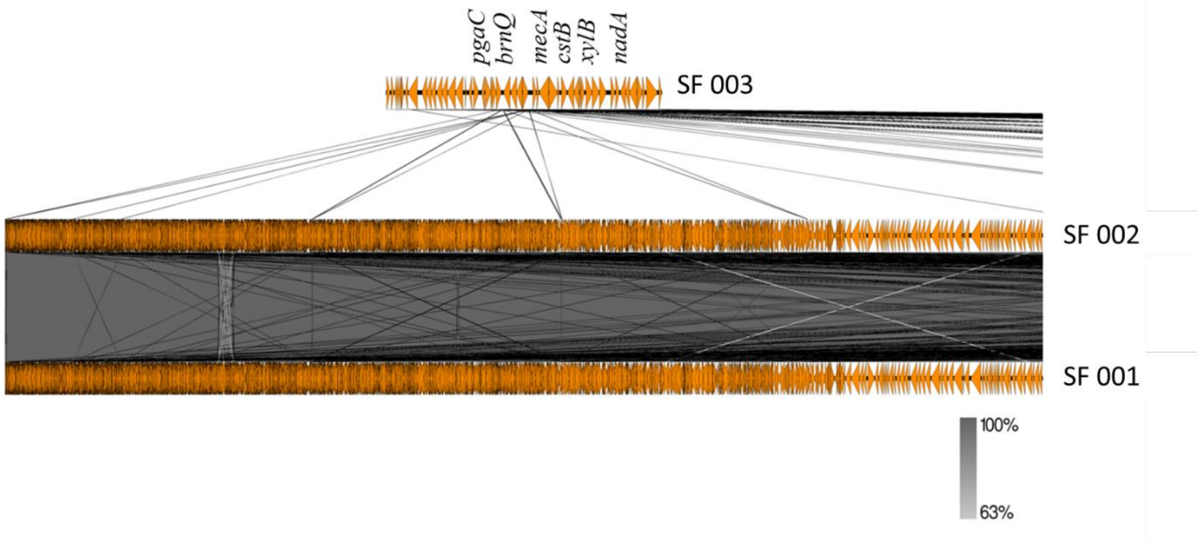

Figure S2: Genomic organization of the *mecA* gene locus of each *Mammaliicoccus fleurettii* isolate (SF001, SF002, and SF003) in this study and comparison of the locus-containing *mecA* gene complex among the isolates. Arrows indicate the translation orientation of the coding genes (EasyFig (4)).

References

1. Martineau F, Picard FJ, Brenier L, Roy PH, Ouellette M, Bergeron MG. 2000. Multiplex PCR assays for the detection of clinically relevant antibiotic resistance genes in staphylococci isolated from patients infected after cardiac surgery. The ESPRIT Trial. J Antimicrob Chemother 46(4):527-534. doi: 10.3201/eid1202.050962
2. Mellman A, Becker K, von Eiff C, Keckevoet U, Schumann P, Harmsen D. 2006. Sequencing and staphylococci identification. Emerg Infect Dis 12(2):333-336. doi: 10.1093/jac/46.4.527
3. Brakstad OG, Aasbakk K, Maeland JA. 1992. Detection of *Staphylococcus aureus* by polymerase chain reaction amplification of the *nuc* gene. J Clin Microbiol 30(7):1654-1660. doi: 10.1128/jcm.30.7.1654-1660.1992
4. Sullivan MJ, Petty NK, Beatson SA. Easyfig: a genome comparison visualizer. Bioinformations. 2011; 27(7): 1009-1010. doi: 10.1093/bioinformatics/btr039
